# The effect of differentiation and TGFβ on mitochondrial respiration and mitochondrial enzyme abundance in cultured primary human skeletal muscle cells

**DOI:** 10.1038/s41598-017-18658-3

**Published:** 2018-01-15

**Authors:** Christoph Hoffmann, Selina Höckele, Lisa Kappler, Martin Hrabĕ de Angelis, Hans-Ulrich Häring, Cora Weigert

**Affiliations:** 1 Division of Pathobiochemistry and Clinical Chemistry, Department of Internal Medicine IV, University Hospital Tübingen, Tübingen, Germany; 2 Institute of Experimental Genetics, Helmholtz Zentrum München, German Research Center for Environmental Health (GmbH), Neuherberg, Germany; 3 German Center for Diabetes Research (DZD), Neuherberg, Germany; 4 Chair of Experimental Genetics, Center of Life and Food Sciences Weihenstephan, Technische Universität München, Freising-Weihenstephan, Germany; 5 Institute for Diabetes Research and Metabolic Diseases of the Helmholtz Zentrum München at the University of Tübingen, Tübingen, Germany

## Abstract

Measuring mitochondrial respiration in cultured cells is a valuable tool to investigate the influence of physiological and disease-related factors on cellular metabolism; however, the details of the experimental workflow greatly influence the informative value of the results. Working with primary cells and cell types capable of differentiation can be particularly challenging. We present a streamlined workflow optimised for investigation of primary human skeletal muscle cells. We applied the workflow to differentiated and undifferentiated cells and we investigated the effect of TGFβ1 treatment. Differentiation of myoblasts to myotubes increased mitochondrial respiration and abundance of mitochondrial enzymes and mitochondrial marker proteins. Differentiation also induced qualitative changes in mitochondrial protein composition and respiration. TGFβ1 reduced complex IV protein MTCO1 abundance in both myoblasts and myotubes. In myoblasts, spare electron transport system (ETS) capacity was reduced due to a reduction in maximal oxygen consumption. In TGFβ1-treated myotubes, the reduction in spare ETS capacity is mainly a consequence of increased oxidative phosphorylation capacity and complex III protein UQCRC2. Taken together, our data shows that it is important to monitor muscle cell differentiation when mitochondrial function is studied. Our workflow is not only sensitive enough to detect physiological-sized differences, but also adequate to form mechanistic hypotheses.

## Introduction

Skeletal muscle is by far the biggest organ of the human body, accounting for roughly 40% of body mass in non-obese individuals and is responsible for 22% of basic metabolic rate^1^. In extreme cases of exercise or work, muscle activity can more than triple the daily energy expenditure^2^. It plays a central role in energy homeostasis, metabolism and metabolism-related diseases. Skeletal muscle is responsible for more than 85% of insulin-dependent glucose uptake in healthy subjects^3^. Peripheral insulin resistance in skeletal muscle leads to reduced postprandial glucose clearance and is considered the starting point for the development of type 2 diabetes^4,5^. It is accompanied by ectopic lipid accumulation and mitochondrial dysfunction^6,7^. Targeting mitochondrial function with drugs or exercise interventions is a cornerstone in the prevention of type 2 diabetes^8–11^. Mitochondria play a central role in energy metabolism and the link between metabolic diseases, including diabetes, and muscle mitochondrial function is well described^12^. Mitochondria house the citric acid cycle, β-oxidation and the respiratory chain and are the source of oxidative ATP generation. Furthermore, they play a role in cellular stress response and apoptosis^13^. With insulin resistance and type 2 diabetes being on a constant rise, it is mandatory to understand skeletal muscle energy metabolism and its regulation.

Primary human skeletal muscle cells obtained from *vastus lateralis* biopsies can be differentiated in culture from myoblasts to multinucleated myotubes and are a widely used system to study regulation of muscle metabolism and mitochondrial function on a molecular level^14–17^. Most researchers use the differentiated state, as it better mimics the *in vivo* situation in muscle^18^. However, the in-culture differentiation process almost never results in 100% differentiated cells and many experimental conditions and secretion factors can influence the differentiation efficiency^19,20^. Effects on differentiation of myoblasts to myotubes can both mask or feign the effects of the treatment on mitochondrial function or metabolic regulators. Thus, it is necessary to tell apart the response of myoblasts and myotubes to the treatment of interest and to carry out additional experiments using myoblasts in order to exclude secondary effects stemming from mixed cultures or differentiation effects. Here, we describe a workflow for assaying the influence of candidate factors on mitochondrial respiration and enzyme content in differentiated and undifferentiated primary human skeletal muscle cells. We report optimised culture conditions that enhance the percentage of differentiated cells, a modified SUIT- (Substrate-Uncoupler-Inhibitor Titration) protocol and an immunoblot strategy to support the elucidation of underlying mechanisms. We describe the difference between the differentiated and undifferentiated cells and as an example we interrogate the influence of TGFβ1 on mitochondrial respiration and abundance of key mitochondrial enzymes in differentiated and undifferentiated cells. TGFβ is of interest since it is a candidate believed to influence mitochondria in muscle *in vivo*^21^. TGFβ can be released from macrophages locally in muscle^22^ and reduces abundance of PGC1α^23^, a master regulator of energy metabolism and mitochondrial biogenesis^24–26^. TGFβ inhibits muscle differentiation^27^, reduces insulin action in primary human myotubes^28^ and blockage of TGFβ action protects from diabetes and obesity^29^. Thus, TGFβ1 treatment serves as an example to demonstrate that our workflow is suitable for uncovering small differences in mitochondrial respiration and enzyme abundance in human skeletal muscle cells and can be successfully employed to elucidate the underlying mechanisms.

## Results

### Removal of FBS during differentiation reduces number of non-fused cells

**Figure 1:**
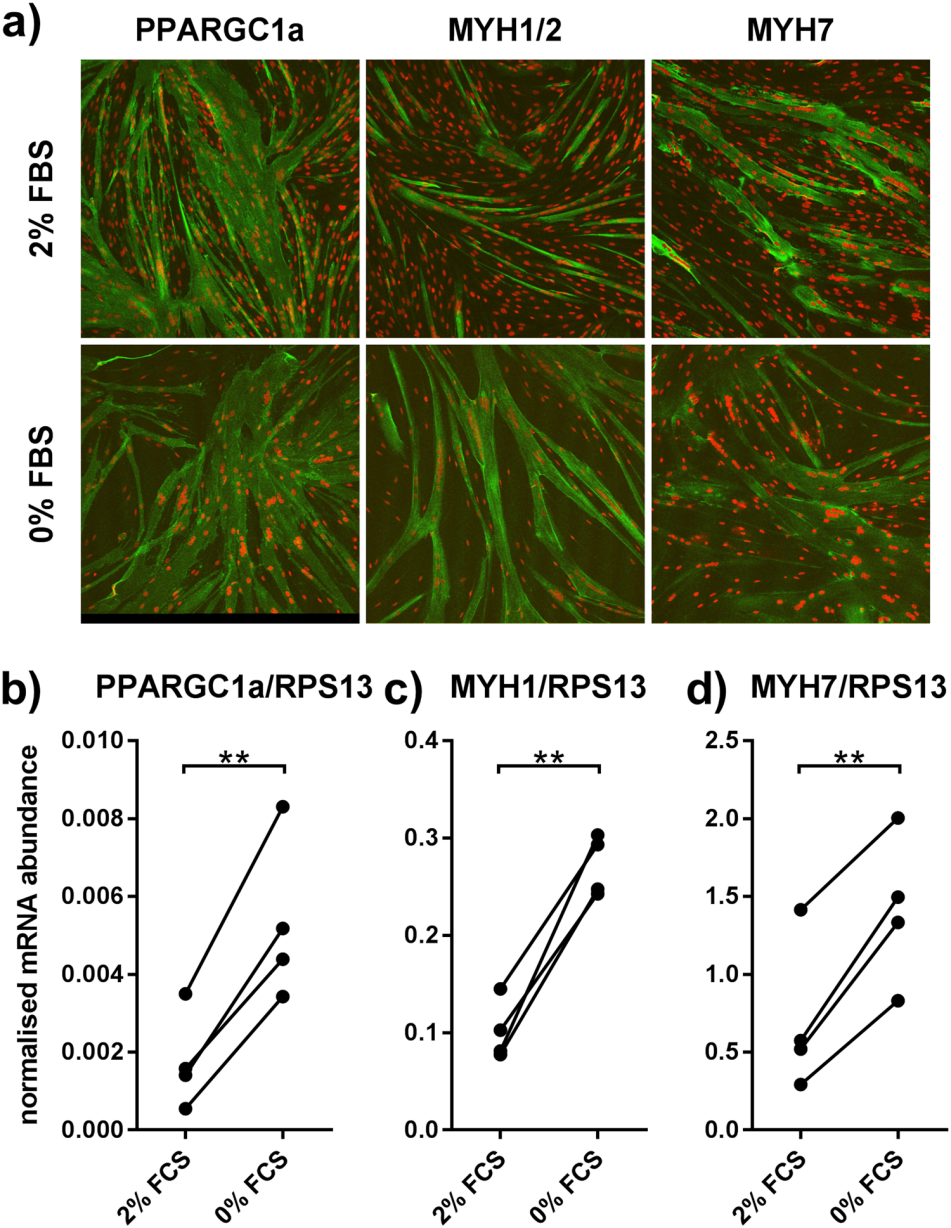
Omitting serum during differentiation reduces the number of non-fused cells. Myoblasts were grown to subconfluence and subsequently differentiated for five days in medium with 2% FBS or without FBS. (**a**) Cells were subsequently stained for skeletal muscle myosin (green, MYH7 or MYH1 + 2) or CD56 (green) and TOPRO (red, nuclei) and images were obtained at 100x magnification. (**b–d**) mRNA abundance of PPARGC1A, MYH1 and MYH7 related to RPS13 as measured by qPCR. Lines indicate points belonging to the same biopsy. Two-sided, paired t-test. n = 4; **p < 0.01.

Cell cultures obtained from human muscle biopsies contain two major cell types: CD56^+^, TE7^−^ myoblasts and CD56^−^, TE7^+^ fibroblasts. To ensure a high concentration of myoblasts, we combine three techniques: Firstly, culturing cells on GelTrex coated dishes increases growth of myoblasts. Secondly, beads coated with an anti-CD56 antibody are used to enrich myoblasts. Thirdly, myoblast content is checked for every batch of cells by flow cytometry. The average content of CD56+ cells used in this study was 95% (Figure S1). Human myoblasts can be used in two states: undifferentiated and differentiated. Differentiated cells are closer to the *in-vivo* situation of myofibers, but common differentiation protocols yield a mixture of unfused and fused cells, making data interpretation complicated. To facilitate a culture consisting primarily of differentiated myotubes, we replaced the FBS (2% final) in the differentiation medium by BSA complexed with free fatty acids and carnitine to compensate for absence of protein in medium and to better mimic physiological nutrient supply. This led to a reduced number of non-fused cells between myotubes (Fig. 1a) and relative mRNA abundance of PGC1α (gene name PPARGC1a) and of differentiation markers MYH1 and MYH7 increased 3.8-fold, 2.8-fold and 2.4-fold, respectively (Fig. 1b). Using this protocol, optimal differentiation was achieved after 5 days.

### Differentiation changes both quantity and quality of mitochondrial respiration

**Figure 2:**
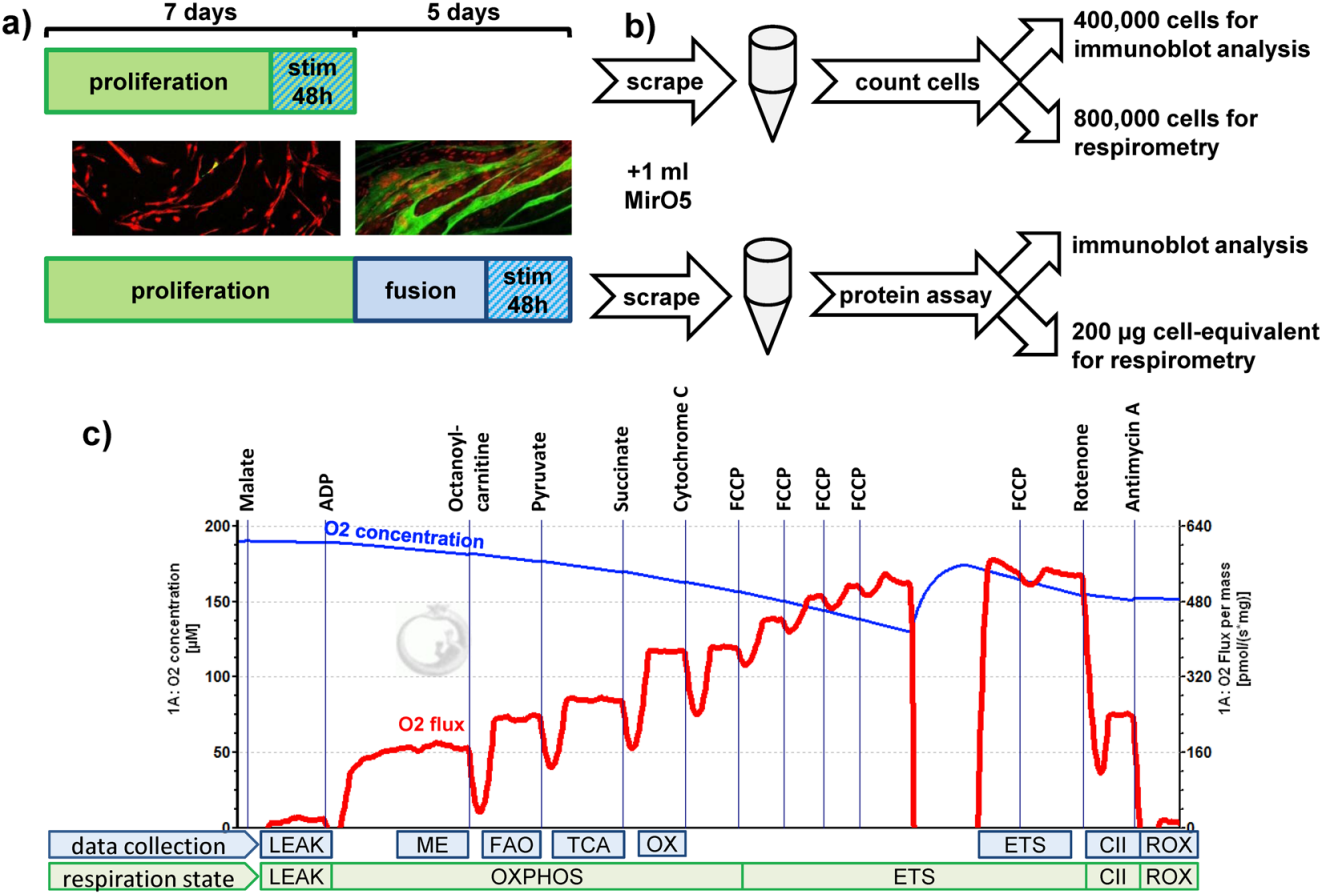
Workflow for characterisation of mitochondrial function using high resolution respirometry and immunoblot. (**a**) Myoblasts were seeded on 15 cm dishes and after 80–90% subconfluence differentiated for five days to myotubes. Stimulation with TGFβ1 was performed two days before subconfluence or the last two days of differentiation. (**b**) Cells were collected by scraping in 1 ml MirO5. Myoblasts were counted and 400,000 cells were collected for protein extraction while 800,000 cells were used for respirometry. For myotubes, 400 µl scraped lysate was pelleted and protein was extracted and quantified. A volume containing 200 µg cellular protein was then used for respirometry. Protein extracts were used for immunoblot analysis. (**c**) High respiration respirometry of myoblasts and myotubes was carried out using the shown injection order. A representative respiration measurement of myotubes, normalised to mg protein, is shown. Different states of respiration are calculated. The states indicated at “data collection” denote where the measurements reported in this manuscript were obtained. LEAK: respiration corresponding to proton leak. ME: malic-enzyme dependent respiration. FAO: fatty acid oxidation, increase after addition of octanoylcarnitine. TCA: respiration, after addition of all substrates that need to pass through the tricarboxylic acid cycle. OX: oxidative phosphorylation (OXPHOS) after addition of succinate and all TCA substrates. ETS: maximal capacity of the electron transport system after uncoupling. CII: complex II respiration after blocking complex I by rotenone addition. ROX: non-mitochondrial/residual respiration after blocking complex III with antimycin A.

We measured respiration in permeabilised primary human myoblasts and myotubes using high resolution respirometry as described in Fig. 2. For myoblasts we used 800,000 cells. For myotubes, accurate counting is difficult due to their multinucleated state and variable size. Therefore, we used scraped cell solution corresponding to 200 µl protein content (Fig. 2b). Cultured myoblasts and myotubes both are able to respire with ADP and malate alone (Fig. 2c), most likely due to the presence of malic enzyme (ME). The commonly used order malate, octanoylcarnitine, ADP thus makes it impossible to dissect fatty acid oxidation (FAO) from malate/ME respiration and was changed to malate, ADP, octanoylcarnitine. FAO is equal to the increase in respiration after addition of octanoylcarnitine. FAO can only be assessed correctly if the addition of pyruvate after octanoylcarnitine increases respiration; otherwise, FAO is limited by the saturated TCA cycle.

**Figure 3:**
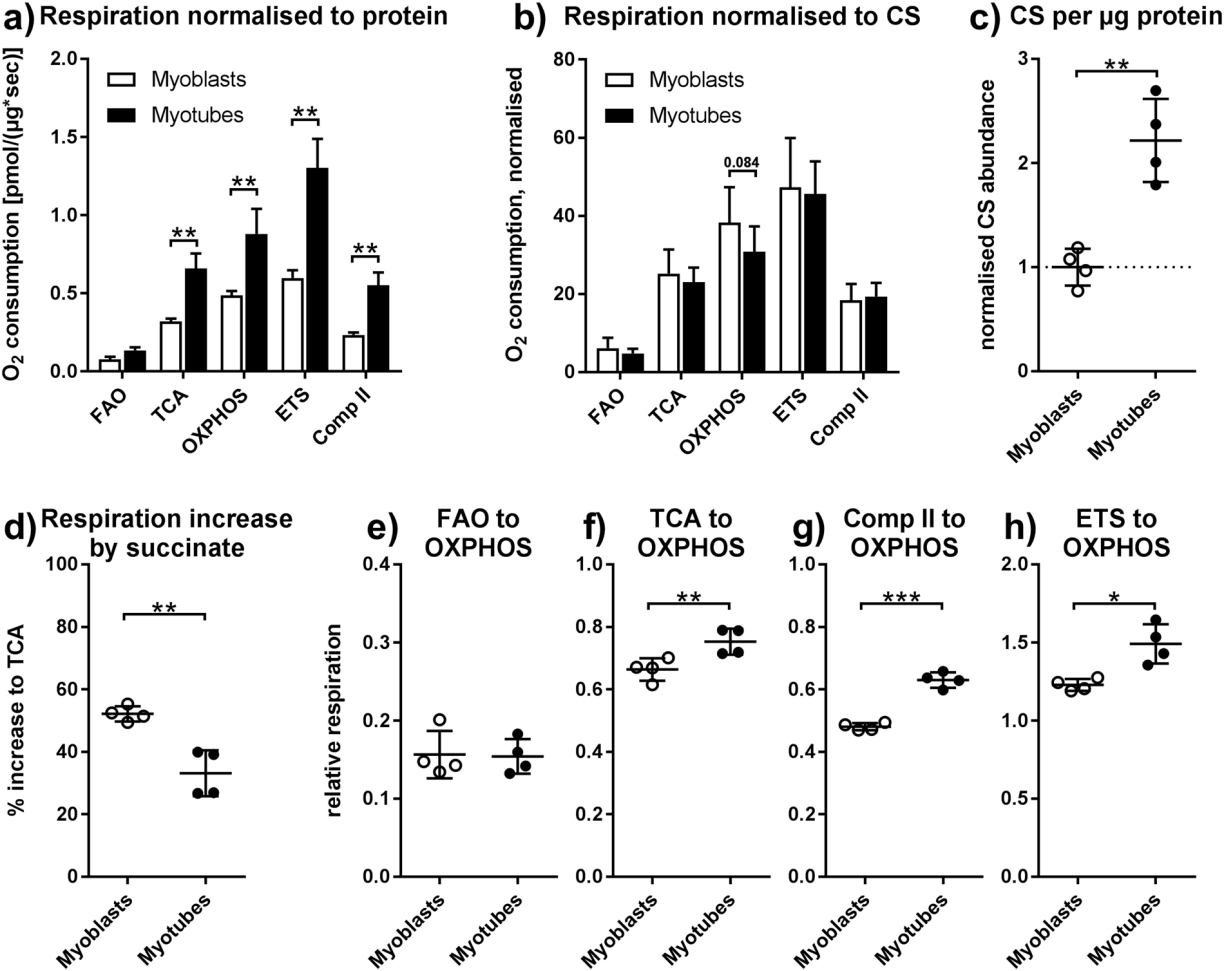
Mitochondria in myotubes are more abundant and qualitatively different from mitochondria in myoblasts. Oxygen consumption of myoblasts and myotubes was measured according to the scheme in Fig. 2. Shown are only cells without TGFβ1 treatment. (**a**) Respiration normalised to total protein content post measurement. (**b**) Respiration normalised to total CS content post measurement. (**c**) CS content normalised to total protein content, both after measurement, and standardised to myoblasts. (**d**) Relative increase in respiration by succinate addition (**e**) β-oxidation respiration relative to OXPHOS capacity. (**f**) Respiration after addition of pyruvate relative to OXPHOS capacity. (**g**) Respiration after addition of rotenone relative to OXPHOS capacity. (**h**) Maximal FCCP respiration relative to OXPHOS capacity. (**a,b**) Repeated measures two-way ANOVA. (**c** to **h**) Paired, two-sided t-tests. *p < 0.05, **p < 0.01, ***p < 0.01. n = 4, mean ± SD.

On average, respiration was 2-fold higher in myotubes than in myoblasts when normalised to protein content, reaching a maximal ETS respiration of 1.30 and 0.6 pmol O_2_/sec per μg protein, respectively (Fig. 3a). The lowest difference was seen in FAO and OXPHOS-respiration (1.83 fold) while the highest difference was seen in ETS and complex II respiration (2.21 and 2.38-fold). TCA-respiration was 2.08-fold higher in myotubes. The differences in respiration rates largely disappear when normalising to citrate synthase (CS) content, with a tendency toward a lower OXPHOS capacity per CS abundance in myotubes. This indicates that a large part of the difference is due to increased mitochondrial content of myotubes (Fig. 3b). Indeed, the amount of CS per protein is 2.24-fold higher in myotubes (Fig. 3c). The lower increase in OXPHOS capacity stems from a reduced increase (36.4% lower) in respiration after succinate addition in myotubes (Fig. 3d). No difference was seen in TCA respiration, meaning maximal non-uncoupled respiration that can by achieved with substrates that feed acetyl-CoA into the citric cycle. We next calculated flux control ratios to elucidate qualitative differences between myoblasts and myotubes (Fig. 3e–h). Compared to total maximal OXPHOS capacity, myotubes show an increase in TCA-driven respiration (13.4%), in complex II respiration (31.1%) and in ETS respiration (21.3%). Relative FAO-respiration was not different. The differences in flux control ratios disappear when compared to ETS capacity instead of OXPHOS capacity.

### Differentiation leads to increased mitochondrial content and enzyme abundance

**Figure 4:**
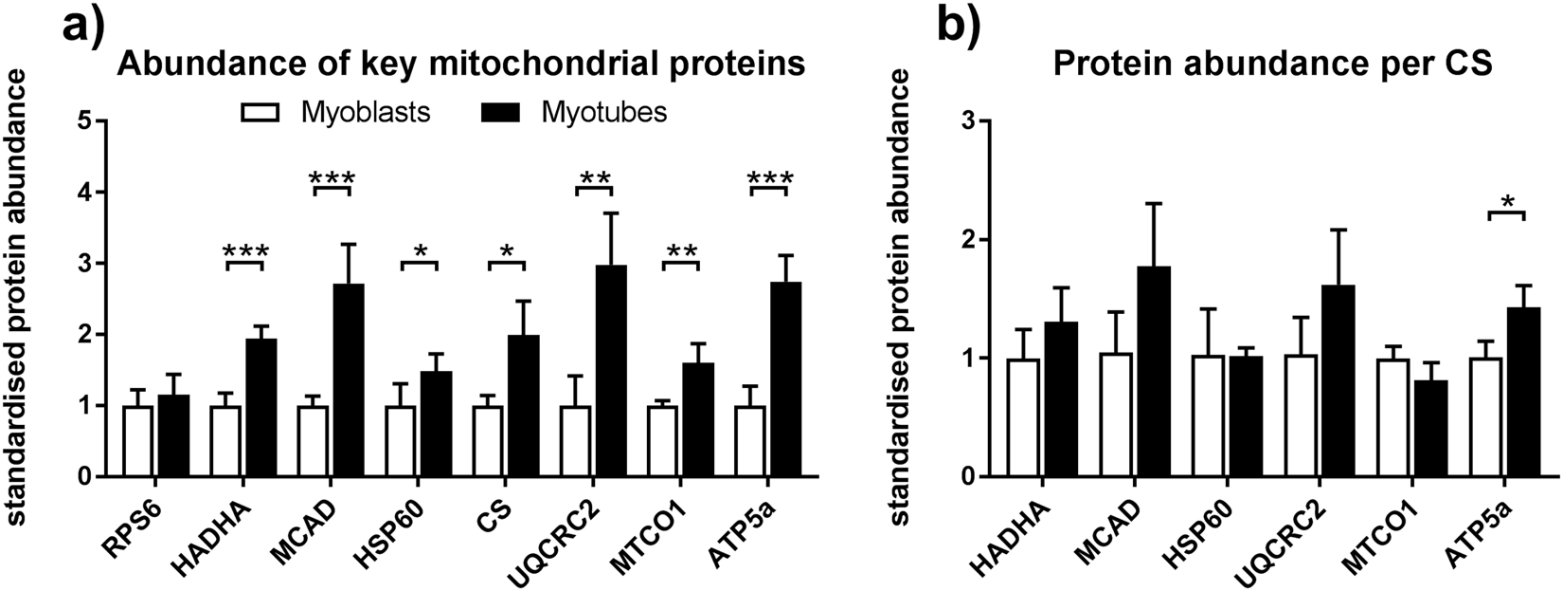
Myotubes contain more mitochondria-specific proteins than myoblasts. Proteins were extracted from myoblasts and myotubes and characterised by immunoblot according to Fig. 2. Only pre-respirometry samples are shown. (**a**) Abundance normalised to protein loaded of mitochondrial abundance markers (CS and HSP60), respiratory chain complex enzymes (UQCRC2, MTCO1 and ATP5), β-oxidation enzymes (HADHA and MCAD), and RPS6 as a housekeeping protein (**b**) Abundance normalised to CS. Only vehicle-treated cells are shown. All values were standardised to myoblasts protein abundances. Two-sided t-tests, *p < 0.05, **p < 0.01, ***p < 0.001. n = 3–4, mean ± SD. Due to missing CS values, less data points could be included for (**b**) than for (**a**).

To investigate mitochondrial enzyme content we used the sample aliquots collected for protein analysis before measurement. When normalised to total protein content, myotubes contain more mitochondrial abundance markers (98.6% more CS, 48.3% more HSP60), respiratory chain complex marker enzymes (UQCRC2 (complex III), MTCO1 (complex IV) and ATP5 (complex V): 198%, 60.0% and 174% more) and β-oxidation enzymes (HADHA and MCAD: 94.2% and 172%) than myoblasts (Fig. 4a). To elucidate whether there is a difference in abundance of key metabolic enzymes in the average mitochondrion, we also normalised our data to CS abundance. The difference in ATP5 remained significant (42.3%) (Fig. 4b) and there is a trend toward higher UQCRC2 (57.3%) and MCAD (69.6%) in myotubes.

### TGFβ1 reduces respiratory spare capacity

**Figure 5:**
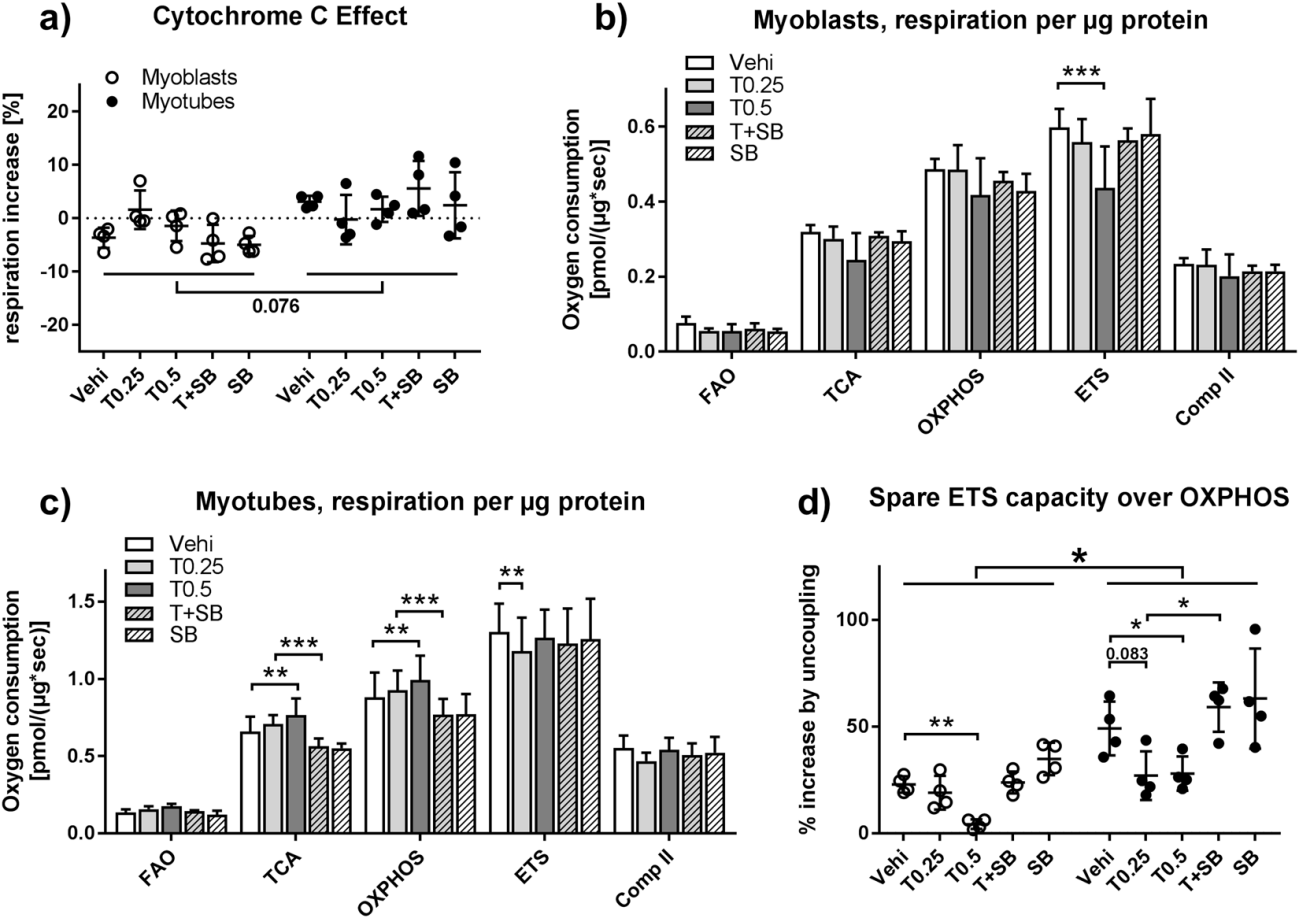
TGFβ1 influences respiratory chain activity and spare capacity in myoblasts and myotubes. Primary human myoblasts and myotubes were treated with TGFβ1 (0.25 or 0.5 ng/ml) or SB431542 (10 μM) for 48 hours and processed according to Fig. 2 and high resolution respirometry was carried out. (**a**) Cytochrome C effect calculated by dividing respiration after addition of cytochrome C by respiration after succinate addition. (**b**) Respiration rates after the indicated additions in myoblasts and (**c**) myotubes. (**d**) Spare respiratory capacity calculated by dividing the difference between ETS and OXPHOS capacity by OXPHOS capacity. For (**a** and **d**) one-way ANOVA was performed for each differentiation state. For (**b** and **c**) two-way ANOVA was performed. *p < 0.05, **p < 0.01. ***p < 0.001. n = 3–4, mean ± SD.

To investigate how TGFβ1 influences mitochondrial respiration we treated both myoblasts and myotubes with TGFβ1 and the TGFβ type I receptor inhibitor SB431542 for 48 h before analysis. TGFβ1 treatment had no effect on mitochondrial intactness as measured by the cytochrome C addition during respirometry. Myotubes showed a tendency toward a slightly higher cytochrome C effect (Fig. 5a). In myoblasts, treatment with 0.5 ng/ml TGFβ1 led to a 26.9% reduction in maximal ETS capacity (Fig. 5b). In myotubes, TGFβ1 treatment led to an increase in TCA (16.3%) and OXPHOS (12.8%) capacity, with only a minor effect on ETS capacity (Fig. 5c). Calculating the spare ETS capacity over OXPHOS capacity reveals that TGFβ1 treatment reduced spare ETS capacity in both myoblasts and myotubes. This was caused by the reduction in ETS capacity in myoblasts and by the increase in OXPHOS capacity in myotubes. In myoblasts, the reduction is 17% for 0.25 ng/ml of TGFβ1 and 81.4% for 0.5 ng/ml TGFβ1, while in myotubes the differences are 45% and 43.1%. The effect disappeared after addition of SB431542. Finally, spare ETS capacity is significantly higher in myotubes than in myoblasts (Fig. 5d).

### Reduced MTCO1 abundance is the most likely explanation for reduced ETS capacity in myoblasts

**Figure 6:**
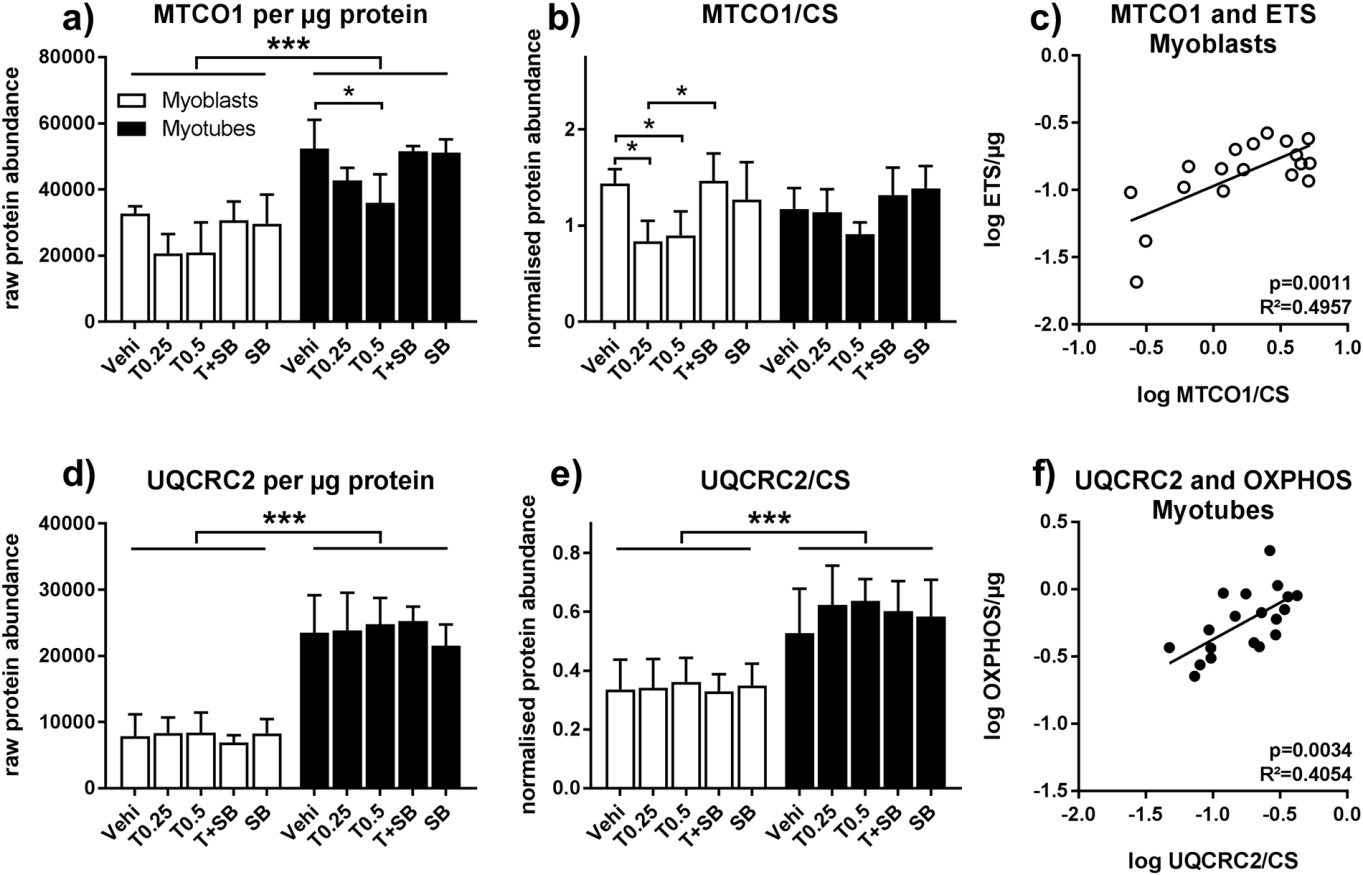
TGFβ1 modifies respiration in myoblasts by influencing MTCO1. Primary human myoblasts and myotubes were treated with TGFβ1 (0.25 or 0.5 ng/ml) or SB431542 (10 μM) for 48 hours and processed according to Fig. 2. Pre-respirometry protein samples were analysed by immunoblot. (**a**) MTCO1 abundance per mg protein loaded. (**b**) MTCO1 normalised to CS abundance. (**c**) Correlation between MTCO1/CS and ETS capacity (Fig. 4c) in myoblasts. (**d**) UQCRC2 abundance per µg protein loaded. (**e**) UQCRC2 normalised to CS abundance. (**f**) Correlation between UQCRC2/CS and ETS capacity (Fig. 4d) in myotubes. (**a**,**b**,**d,e**) Two-way ANOVA, *p < 0.05, ***p < 0.001, n = 3–4, mean ± SD. (**c**,**f**) Linear regression modelling using log-transformed data. Representative blot examples can be found in Figure S2.

To elucidate the mechanism of how TGFβ1 treatment influences respiration, we quantified abundance of mitochondrial enzymes and marker proteins in pre-measurement samples. In both myoblasts and myotubes there was a tendency toward lower MTCO1, a marker for complex IV abundance, both when normalised to protein loaded and CS. The trend reached significance for MTCO1/protein in myotubes treated with 0.5 ng/µl TGFβ1 (31.2% reduction, Fig. 6a) and for MTCO1/CS in myoblasts treated with 0.25 and 0.5 ng/ml TGFβ1 (41.8% and 37.5% reduction, Fig. 6b). In both cases, the effect is gone when SB431542 is added in parallel with TGFβ. In myoblasts, MTCO1/CS and MTCO1/protein positively correlates with ETS capacity, supporting a causal relationship between reduced ETS capacity and reduced MTCO1 abundance (Fig. 6c and Table S1). ATP5 also correlates with ETS and OXPHOS capacity in myoblasts (Table S1). For myotubes, no further TGFβ1 effects were detected using immunoblot (Fig. 6d,e and Figure S2), but the pattern seen for UQCRC2 abundance matches the pattern seen for OXPHOS capacity. Indeed, UQCRC2, at least when normalised to CS, correlates with OXPHOS capacity (Fig. 6f). Notably, the increase in UQCRC2, MCAD and HADHA in myotubes, compared to the myoblasts seen in Fig. 4, reaches significance even after normalisation to mitochondrial protein content when including all stimulation conditions (Fig. 6e and Figure S2).

### TGFβ1 signalling influences differentiation of myotubes and MTCO1

**Figure 7:**
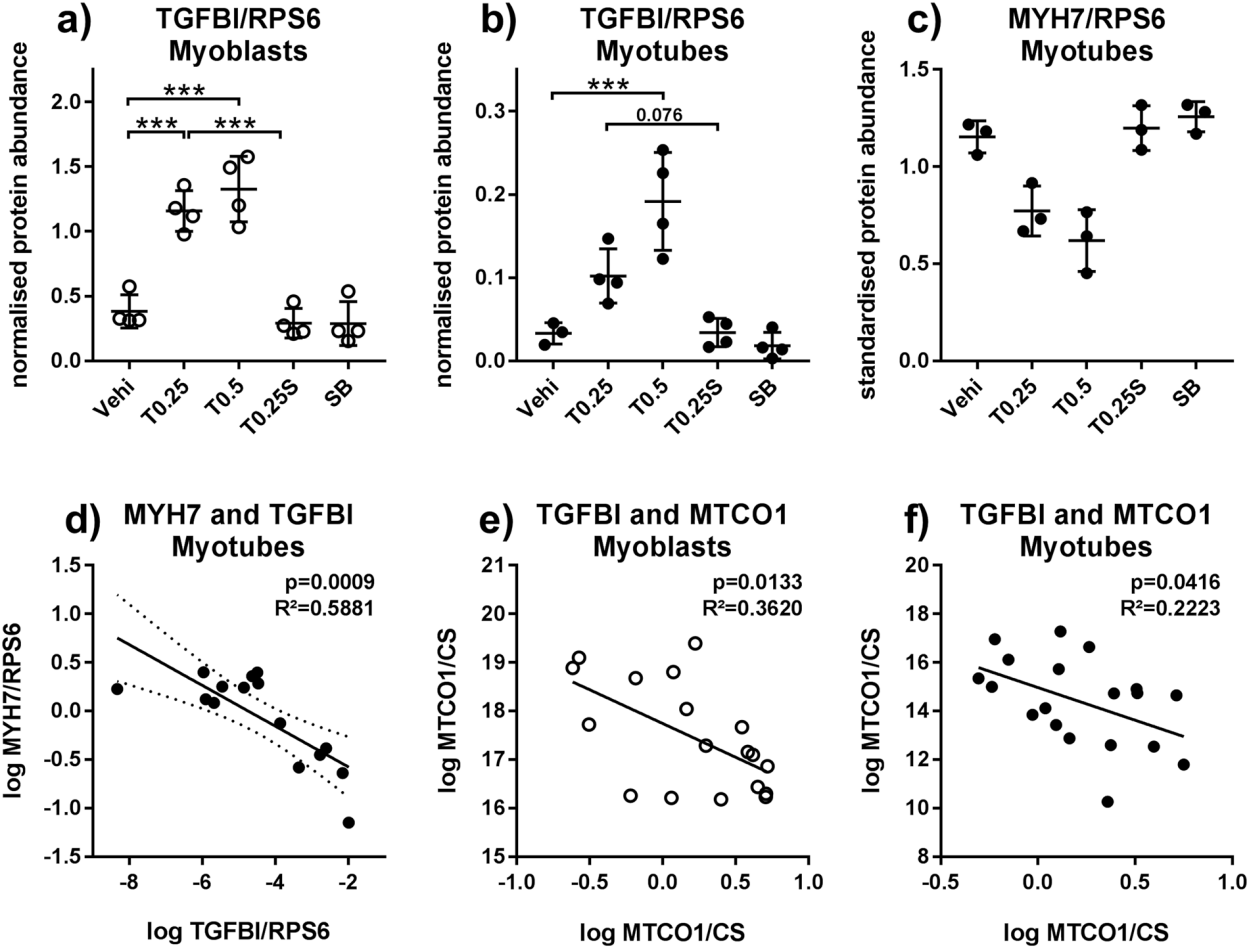
TGFβ1 influences differentiation and MTCO1 abundance. Primary human myoblasts and myotubes were treated with TGFβ1 (0.25 or 0.5 ng/ml) or SB431542 (10 μM) for 48 hours and processed according to Fig. 2. Pre-measurement samples were analysed by immunoblot. (**a** and **b**) TGFBI, normalised to RPS6. (**c**) MYH7, normalised to RPS6 and standardised for donor. (**d**) Correlation of TGFBI (normalised, log-transformed) and MYH7 (normalised, standardised, log-transformed) in myotubes. (**e**,**f**) Correlation of TGFBI and MTCO1/CS in myoblasts and myotubes. For (**a** to **c**) one-way ANOVA and for (**d** to **f**) linear regression modelling was used. *p < 0.05, **p < 0.01. ***p < 0.001. n = 4 for TGFBI, n = 3 for MYH7, mean ± SD. For unknown reasons, the pre-measurement samples for one donor gave no MYH7 signal, despite the post-measurement samples doing so.

We used immunoblot to measure TGFBI (TGFβ inducible protein) abundance as a marker for activation of TGFβ pathways and MYH7 to assess the influence of TGFβ1 on differentiation. No MYH7 protein was detected in myoblasts and myoblasts contained more TGFBI protein than myotubes (Figure S2a). TGFβ1 treatment increased TGFBI abundance in both myoblasts and myotubes between 3-fold in myoblasts (Fig. 7a) and 5.8-fold in myotubes (Fig. 7b). There was a trend toward lower MYH7 abundance in TGFβ1 treated myotubes, being 33% lower for 0.25 ng/µl and 46% lower for 0.5 ng/ml (Fig. 7c), but the trend did not reach significance. The effects were absent when SB431542 is added in parallel with TGFβ. Still, there was a negative correlation between TGFBI and MYH7 (Fig. 7d), well in line with TGFBI being a marker for active TGFβ1 signalling^30^ and TGFβ1 impairing differentiation, thus supporting a causal relationship. Notably, abundance of TGFBI showed a negative correlation with MTCO1 in both myoblasts and myotubes (Fig. 7e,f) underlining the negative effect of activated TGFβ1 signalling on MTCO1, as depicted in Fig. 6a,b.

## Discussion

Here, we report an optimised workflow, applying high-resolution respirometry and immunoblot-characterisation to interrogate mitochondrial energy metabolism. We pay special attention to the experimental design before and after respiratory, covering all steps from seeding to hypothesis generation. To improve quality of sample material, we modified previously described techniques. A factor that favours the proliferation of CD56^+^ myoblasts and reduces the number of e.g. fibroblasts is coating cell culture dishes with extracellular matrix components^31,32^. We enriched CD56^+^ myoblasts by culturing on GelTrex-coated vessels and by purification using anti-CD56 magnetic beads. Using this procedure, cell purity reached in average above 90% CD56^+^ cells, exceeding the content achieved by coating and optimised cell culture media alone^31^ and well in line with previous data^33^. We further enriched the number of differentiated myotubes by omitting FBS during differentiation to yield a culture containing a lower amount of non-fused cells and higher differentiation marker abundance. We suggest that fused myotubes tolerate the absence of FBS in culture while non-fused cells do not. During respirometry, we found that it is crucial to inject ADP between malate and octanoylcarnitine in order to to distinguish between fatty acid oxidation and respiration driven by malic enzyme released pyruvate. Mitochondrial malic enzyme in skeletal muscle is often ignored, despite the fact that malic enzyme activity has been described in several cell lines, including C2C12^34^. The main reason for that is that only cytosolic malic enzyme (ME1) is expressed^35^ in skeletal muscle tissue and that malic enzyme activity is absent in permeabilised muscle fibres obtained directly from biopsies. We also recommend recovering chamber content after measurement to normalise respirometry data. For immunoblot analysis we use aliquots from the very same sample that is used for respirometry. Lastly, myoblasts and myotubes should both be interrogated to tell apart primary effects from differentiation effects. That way, we can assay differentiation, effect of treatment, mitochondrial abundance and mitochondrial enzyme content and directly correlate the results to respirometry data.

Using this strategy, we measured the effect of differentiation on mitochondria. Differentiation increases mitochondrial abundance markers, fatty acid oxidation enzymes and respiratory chain enzymes as well as respiration per protein. Our data, obtained from primary human myotubes and myoblasts, is in line with data of Moyes *et al*.^36^, who performed enzyme assays and investigated mitochondrial ultrastructure for several individual enzymes in mouse C2C12 cells and Remels *et al*.^37^, who also measured OXPHOS protein abundance and respiration in C2C12 myoblasts and myotubes. Furthermore, Fortini *et al*. demonstrated an increase of mtDNA in primary mouse myoblasts during differentiation when compared to nuclear DNA^38^. This regulation is partially accomplished by myogenesis-induced miR-1 that stimulates the translation of mitochondrial encoded proteins and enzymes^39^. Other relevant factors that increase during differentiation are TFAM and PGC1α^37,40^. The increased respiration in myotubes is largely due to the increased mitochondrial protein content. Beyond that, we demonstrate qualitative differences between mitochondria in myoblasts and myotubes. When normalised for mitochondrial abundance, myotube mitochondria contain more ATP5a, UQCRC2, HADHA and MCAD, which would have suggested increased FAO and OXPHOS capacity per mitochondrion compared to myoblasts. Unexpectedly, there is a tendency toward lower OXPHOS-capacity per mitochondrial abundance in myotubes. Proteins not measured in the present study may act as a bottleneck. This protein may sit downstream of complex II, as the difference appeared after succinate addition, but is not complex II, as rotenone respiration was comparable. Alternatively, it is possible that the increase in ATP5a overestimates the increase in complex V activity or that CS abundance overestimates mitochondrial content, though CS is a well accepted marker for mitochondrial abundance^41^.

Next, we investigated the effect of TGFβ treatment on respiration. The effect of TGFβ is known to be highly tissue-dependent. In skeletal muscle cells TGFβ negatively impacts differentiation^27^, reduces expression of the mitochondrial regulators PGC1α^23^ and TFAM^28^ and negatively correlates with increase of mitochondrial enzymes and abundance after an exercise intervention^28^. Loss of the TGFβ signal transducer SMAD3 promotes mitochondrial biogenesis and function in white adipose tissue of mice^29^. In contrast, TGFβ has activating properties on diverse mitochondrial parameters in fibroblasts^42^, myofibroblasts^43^ and podocytes^44^. The complex regulation of TGFβ bioactivity and signal transduction by several extra- and intracellular effectors and inhibitors is an obvious possible explanation for cell-type-dependent or tissue-specific effects^45,46^. Here, we detected a negative effect of TGFβ on mitochondrial complex IV protein MTCO1 abundance. In myoblasts, the positive correlation of MTCO1 abundance with ETS capacity and a negative correlation with TGFBI abundance demonstrate that TGFβ signalling leads indeed to reduced ETS capacity via reduction of MTCO1 abundance. This is in line with data from lung epithelial cells where TGFβ also had a negative effect on complex IV activity^47^. In myotubes there was only a minor negative effect on ETS capacity and MTCO1 abundance. Instead, TCA and OXPHOS capacity increased, reducing relative spare ETS capacity. This might be due to increased mitochondrial complex III protein UQCRC2 abundance in TGFβ1-treated myotubes. The mechanism responsible for the altered mitochondrial protein content is not yet clear and might involve different pathways in myoblasts and myotubes. TGFβ1 treatment leads to a rapid suppression of PGC1α transcript levels after 4 h in C2C12 myotubes^23^ and after 24 h in human myotubes^28^ which involves a suppressor function of SMAD3 on PGC1α promoter. PGC1α is a key transcriptional coactivator of TFAM that controls transcription of mitochondrial encoded genes such as MTCO1^24^, a process that is also important during skeletal muscle cell differentiation^40^. Thus, the SMAD3-dependent suppression of PGC1α might be responsible for reduced MTCO1 abundance in TGFβ1-treated myoblasts and myotubes. On the other hand, the negative effect of TGFβ on skeletal muscle cell differentiation can also affect mitochondrial protein abundance. We did not see a reduction in ATP5 abundance in TGFβ1-treated myotubes as detected before^28^, most likely due to the shorter stimulation used here. We also observed a weak effect on differentiation marker MYH7, which can be seen as a first hint that suppression of mitochondrial function in TGFβ-treated myotubes is at least partly due to its inhibitory effect on differentiation. *In vivo*, this might translate to a reduced formation of new muscle fibres.

In summary, our optimised workflow allows to obtain reliable data quality and aids identification and understanding of effects and mechanisms. We were able to detect changes in total mitochondrial abundance and relative mitochondrial enzyme content induced by differentiation and TGFβ treatment and to combine and correlate them with respirometry data. The data underlines the relevance of muscle cell differentiation for mitochondrial function, which needs to be considered when factors influencing differentiation are studied. Our workflow may easily be transferred to other cell lines, including primary cells and cells capable of differentiation.

## Material and Methods

### Human subjects and Cell culture

Satellite cells were obtained by percutaneous needle biopsies performed on *vastus lateralis* muscle. Donors were middle-aged, non-obese and normal glucose tolerant males. Cells were released by collagenase digestion and seeded on 15-cm dishes coated with GelTrex (thin layer protocol, 1:300, Life Technologies, Carlsbad, California, USA). After two rounds of proliferation in cloning medium (39% α-MEM, 39% Ham’s F-12, 20% FBS, 1% chicken extract, 100 U/ml penicillin, 100 μg/ml streptomycin and 0.5 μg/ml amphotericin B), CD56-positive myoblasts were enriched and stored in the gaseous phase of liquid nitrogen. For experiments, cells were seeded at 1000 cells/cm² in cloning medium on coated 15 cm dishes. Upon reaching 80–90% confluence, half of the dishes were used for high-resolution-respirometry and protein extraction. The other half was differentiated in FBS-free fusion medium (α-MEM, 100 U/ml penicillin, 100 μg/ml streptomycin, 0.5 μg/ml amphotericin B) for 5 days and then used for respirometry. Fusion medium furthermore contained fatty acids complexed to BSA (final concentrations: 50 μM palmitate, 50 μM oleate, 1.67 mg BSA, 0.05% ethanol and 100 µM carnitine). For the comparison of fusion media, fusion medium with 2% FBS instead of BSA-coupled fatty acids was used. All stimulations were performed for the last 48 hours before respirometry. Human TGFβ1 (R&D Systems, Minneapolis, USA, product 100-B-001) was solved in 4 mM HCl 0.2% BSA while SB431542 (Milteny Biotech, Bergisch Gladbach, Germany) was solved in DMSO.

### Coupling of fatty acids to BSA

Palmitate and oleate were solved in ethanol at 200 mM. 300 µl of the solution were added to 9.7 ml 10% BSA in DPBS and incubated over night at 37 °C in a shaking incubator, yielding a solution containing 6 mM fatty acid and 3% ethanol. Solutions were aliquoted and stored at −20 °C until use. For each fatty acid, a separate stock solution was generated.

### MACS and flow cytometry

Myoblasts were enriched using MACS microbeads and LS-columns (Milteny Biotech) according to the manufacturer’s protocol, with one exception: bead incubation was increased to 30 minutes. Usually, cells from eight 15 cm dishes (10 to 20 million cells) and 3 μl beads per million cells were used per isolation. Samples were taken before isolation, from flow-through and from eluate to assess content of CD56-positive cells by flow cytometry. For flow cytometry, 250,000 cells were washed thrice in 500 μl PBS containing 1% FBS and stained with AlexaFluor488-labeled anti-CD56 antibody or isotype control (Table 1a). After washing three more times, cells were analysed with a FACSCalibur flow cytometer (BD Biosciences). Usually, between 5,000 and 10,000 cells were analysed per sample.

**Table 1:** Antibodies and primers used.

a) 1^st^ Antibody	Applic.	Dilution	Manufacturer	Order No.
CD56-AF488 (B159)Iso-AF488 (MOPC-21)	FC	1:100	BD Biosciences, Franklin Lakes, New Jersey, USA	557699557702
CD56	ICC	1:100	DSHB Iowa City, IA, USA	5.1H11
CS	IB	1:1000	Abcam, Cambridge, UK	ab96600
HADHA	IB	1:500	Abcam, Cambridge, UK	ab54477
HSP60	IB	1:2000	Santa Cruz Biotech, Dallas, Texas, USA	sc1052
MCAD	IB	1:400	Santa Cruz Biotech, Dallas, Texas, USA	sc365448
MYH1 + 2	IB/ICC	1:3000/1:100	Sigma-Aldrich, Deisenhofen, Germany	M4276
MYH7	IB/ICC	1:3000/1:100	Sigma-Aldrich, Deisenhofen, Germany	M8421
Oxphos Cocktail	IB	1:500	Abcam, Cambridge, UK	MS604
RPS6	IB	1:1000	Cell Signaling, Cambridge, UK	2217
TGFBI	IB	1:1000	R&D Systems, Minneapolis, Minnesota, USA	AF2935
**b) 2** ^**nd**^ **Antibody**	**Applic**.	**Dilution**	**Manufacturer**	**Order No**.
GaR-IRD800CW	IB	1:10000	LI-COR Biosciences, Lincoln, NE, USA	926-32211
GaM-IRD700RD	IB	1:10000	LI-COR Biosciences, Lincoln, NE, USA	925-68070
DaG-IRD800CW	IB	1:10000	LI-COR Biosciences, Lincoln, NE, USA	p26-32214
GaM-AF488	ICC	1:250	Invitrogen, Carlsbad, California, USA	A-11001
**c) qPCR Primer**	**Applic**.	**Dilution**	**Manufacturer**	**Order No**.
RPS13	qPCR	1:10	Qiagen, Hilden, Germany	QT02317532
PPARGC1a	qPCR	1:10	Qiagen, Hilden, Germany	QT00095578
MYH1	qPCR	1:10	Qiagen, Hilden, Germany	QT01671005
MYH7	qPCR	1:10	Qiagen, Hilden, Germany	QT00000602

a) First antibodies for epitope detection. b) Second antibodies for fluorescence detection. c) qPCR primers. IB: immunoblot, FC: flow cytometry, ICC: Immunocytochemistry.

### Immunocytochemistry

Cells were grown on X-well slides (Sarstedt, Nümbrecht, Germany) coated with GelTrex and differentiated as described above. After 5 days of differentiation, they were washed with PBS, fixed in PBS containing 4% formaldehyde (pH 7.4) for 20 min, quenched with 150 mM glycine in PBS for 10 min and permeabilised with 0.1% Triton X100 for 2 min. Blocking was performed with 1% NGS, 0.05% Tween 20 in PBS for 30 min. Antibodies recognizing CD56, MYH1 + 2, or MYH7 (Table 1a) were diluted in blocking solution and added for 1 h at room temperature. After washing thrice in PBS, the second antibody (Table 1b), diluted in blocking solution, was added for 2 h. After another wash, nuclei were stained using TO-PRO3 (Invitrogen, Carlsbad, California, USA) before mounting in PermaFluor (Beckman Coulter, Krefeld, Germany). Images were obtained using a DM-IRBE equipped with a TCS-SP and a DCF340FX (Leica, Wetzlar, Germany).

### qPCR

RNA was extracted employing the NucleoSpin miRNA kit (Macherey-Nagel, Düren, Germany) and reverse transcription was carried out with the Transcriptor First Strand Synthesis Kit (Roche, Basel, Switzerland) using 500–1,000 ng total RNA per 20 µl reaction. cDNA was diluted with 80 µl of nuclease free water and 2.5 µl of this dilution was used in a 20 µl qPCR reaction containing 10 µl QuantiFast SYBR Green PCR Mix and 2 µl QuantiTect Primer Assay (Qiagen, Hilden, Germany, Table 1c) on a LightCycler 480 machine (Roche). Standards for PCR were generated by purifying PCR-Product (MinElute PCR Purification Kit, Qiagen) for each primer and generating a 10-fold serial dilution series ranging from 5 pg/µl to 0.5 ag/µl. Standards were always freshly diluted on the day of experiment from a 5 ng/µl stock solution which was stored in aliquots at −20 °C.

### Immunoblot

Scraped cells from before and after respirometry were collected by centrifugation (20,000 g, 4 °C, 5 min) and lysed with 200 μl of RIPA buffer (25 mM Tris pH7.6, 150 mM NaCl, 0.1% SDS, 0.5% NaDOC, 1% Triton X100, containing protease and phosphatase inhibitors). Protein concentration was quantified with BCA-Assay (Pierce Biotechnology, Waltham, Massachusetts, USA). Proteins were separated by sodium dodecyl sulfate polyacrylamide (7.5–15%) gradient gel electrophoresis and transferred onto a PVDF membrane (Immobilon FL, Millipore, Billerica, Massachusetts, USA) by semi-dry electroblotting (transfer buffer: 48 mM Tris, 39 mM glycine, 0.0375% sodium dodecyl sulfate, and 20% (v/v) methanol). Blocking was done in NET buffer (150 mM NaCl, 50 mM Tris/HCl, pH 7.4, 5 mM EDTA, 0.05% Triton X-100, and 0.25% gelatine). The first antibody (Table 1a) was added in NET overnight at 4 °C. After washing with NET buffer, the membranes were incubated with a second first antibody from another species for 2 hours at room temperature. After washing again, fluorescence-labeled secondary antibodies (LI-COR Biosciences, Lincoln, NE, USA, Table 1b) were added (1 h, room temperature). Detection was performed on a Odyssey scanner (LI-COR Biosciences).

### High resolution respirometry

Medium was removed, cells were washed with PBS and put on ice. 1 ml of Mir05 (0.5 mM EGTA, 3 mM MgCl_2_, 60 mM Lactobionic acid, 20 mM Taurine, 10 mM KH_2_PO_4_, 20 mM HEPES, 110 mM D-Sucrose, 1 g/l essentially fatty acid free BSA, pH7.1) buffer was added and cells were collected by scraping. For undifferentiated myoblasts, cells were counted using a haemocytometer. 400,000 cells were pelleted (20,000 g, 5 min, 4 °C) for protein extraction, buffer was completely removed and the pellet was immediately frozen on dry ice. For respirometry, 800,000 cells were pelleted (10,000 g, 5 min, 4 °C), buffer was removed and the cell pellet was resolved in 200 μl of Mir05 using a 500 μl Hamilton syringe. In case of differentiated myotubes, clumps were broken by shearing through a 200 μl pipette tip and 400 μl scraped cell solution was pelleted (20,000 g, 5 min, 4 °C) for protein extraction and BCA assay. The extract was then frozen on dry ice. For respirometry, cell solution corresponding to 200 μg protein by BCA was pelleted (10,000 g, 5 min, 4 °C), buffer was removed and the cell pellet was resolved in 200 μl of Mir05 using a 500 μl Hamilton syringe. For measurement, the resuspended samples were injected into the measurement chamber of an Oxygraph-2k (Oroboros Instruments, Innsbruck, Austria). The chamber was pre-equilibrated with 2 ml Mir05. After briefly waiting for a plateau in respiration to form, 3 μl digitonin (8,1 mM, in DMSO) and 3,2 μl malate (800 mM, in H_2_O) were injected. After forming of a plateau, the following injections were 10 μl ADP (500 mM, H_2_0), 10 μl octanoylcarnitine (100 mM, H_2_0), 10 μl pyruvate (1 M, H_2_0), 10 μl succinate (500 mM, H_2_0), 5 μl cytochrome C (from equine heart, 4 mM, H_2_0), FCCP titration (1 μl steps, 1 mM, DMSO), 1 μl rotenone (1 mM, ethanol), 1 μl antimycin A (5 mM, ethanol), always waiting for a plateau to form after each injection. Glutamate (10 µl, 500 mM, H_2_O) was tested in myoblasts but since it was not different (p = 0.73, FC = 1.009) from pyruvate respiration it was subsequently omitted. Reoxygenation was done around 125 μM O_2_, which was usually during FCCP titration. After measurement, 1.5 ml sample were recovered while stirring to ensure equal distribution. Cell fragments were collected by centrifugation (20,000 g, 4 °C, 5 min), supernatant was removed and pellets were frozen on dry ice. Post-measurement pellets were subjected to protein extraction, BCA assay and immunoblot and total chamber content of protein and CS was calculated.

### Data processing and statistics

FACS data was evaluated with Flowing Software 2.5.1. Respirometry data was obtained using DatLab6 (Oroboros Instruments). Windows were drawn over the plateau phase after each injection and oxygen consumption rates were exported. During FCCP titration, only the highest plateau was used. ROX (after antimycin A injection) was subtracted and the rates were normalised to total chamber protein or CS content. Immunoblots were scanned using the Odyssey scanner (LI-COR Biosciences), analysed with Image Studio Lite (LI-COR Biosciences) and data was exported for processing. qPCR experiments were analysed using the LightCycler480 software. For linear regression, analysis data was log transformed. Data processing was carried out in Excel (Microsoft Corporation, Redmond, Washington, USA). GraphPad Prism (GraphPad Software, Inc.) was used for calculating statistics and creation of graphs.

### Data availability

The datasets generated during and/or analysed during the current study are available from the corresponding author on reasonable request.

### Ethical approval and informed consent

All donors gave informed written consent to the study, which was approved by The Ethical Committee of the Tübingen University Medical Department. All experiments were performed in accordance with relevant guidelines and regulations.

## Electronic supplementary material

Supplemental Figures and Tables
