# Supplementary material for: The effect of differentiation and TGFβ on mitochondrial respiration and mitochondrial enzyme abundance in cultured primary human skeletal muscle cells
Source: Sci Rep. 2018 Jan 15;8:737. doi: 10.1038/s41598-017-18658-3 (PMC5768688; doi:10.1038/s41598-017-18658-3)
Supplement: Supplementary file 1 — Supplemental Figures and Tables [file 41598_2017_18658_MOESM1_ESM.pdf]

Supplemental Information

The effect of differentiation and TGFβ on mitochondrial respiration and mitochondrial enzyme abundance in cultured primary human skeletal muscle cells

Christoph Hoffmann[1], Selina Höckele[2,3], Lisa Kappler[1], Martin Hrabě de Angelis[2,3,4], Hans-Ulrich Häring[1,3,5], and Cora Weigert[1,3,5]

[1]Division of Pathobiochemistry and Clinical Chemistry, Department of Internal Medicine IV, University Hospital Tübingen, Tübingen, Germany
[2]Institute of Experimental Genetics, Helmholtz Zentrum München, German Research Center for Environmental Health (GmbH), Neuherberg, Germany
[3]German Center for Diabetes Research (DZD)
[4]Chair of Experimental Genetics, Center of Life and Food Sciences Weihenstephan, Technische Universität München, Freising-Weihenstephan, Germany
[5]Institute for Diabetes Research and Metabolic Diseases of the Helmholtz Zentrum München at the University of Tübingen, Tübingen, Germany

*To whom correspondence should be addressed:
Cora Weigert, Department of Internal Medicine, University Hospital Tübingen, Otfried-Müller-Str. 10, 72076 Tübingen; phone:++497071 2985670; fax:++497071 295348
cora.weigert@med.uni-tuebingen.de

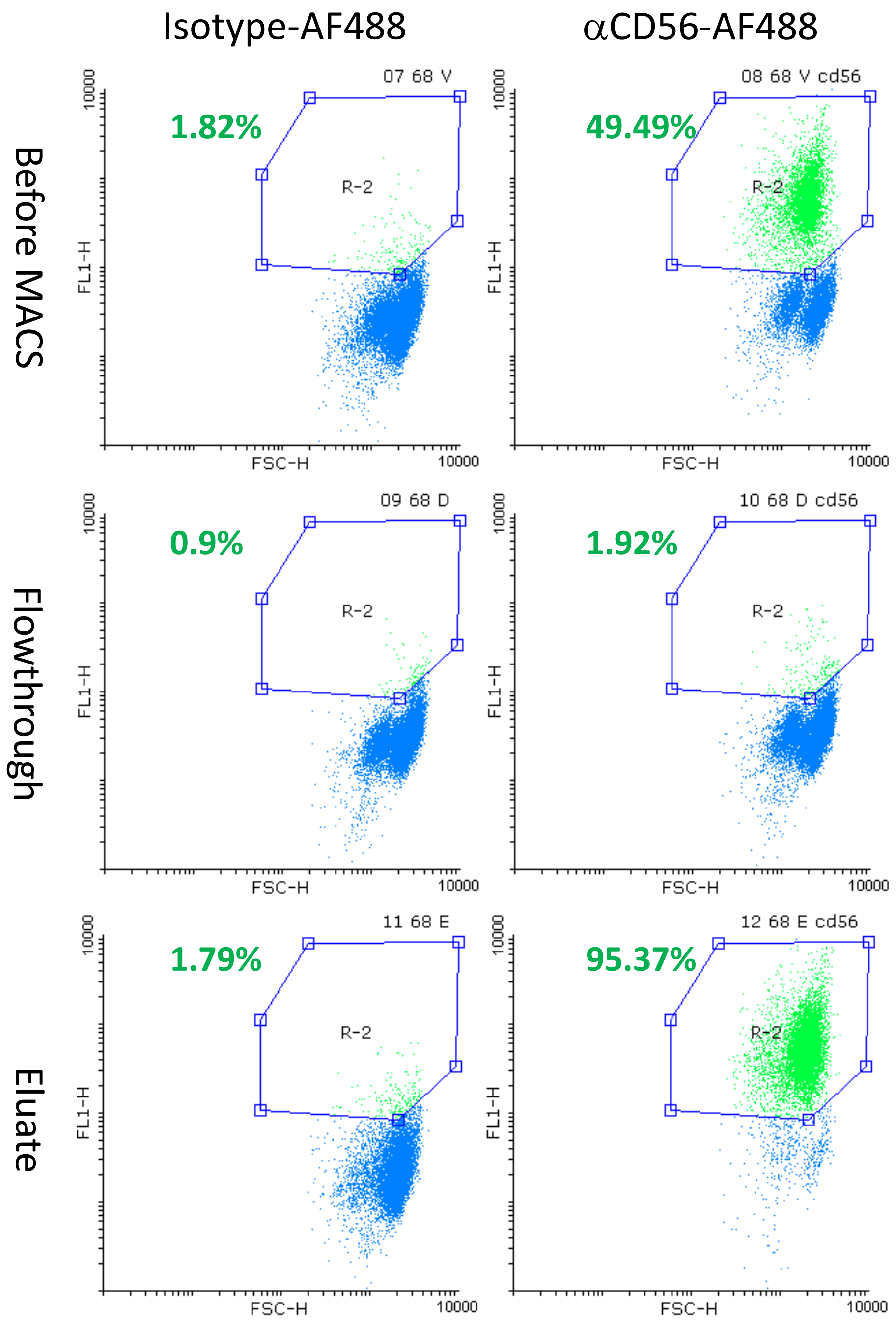

Supplementary Figure S1: Enrichment of myoblasts. Primary human skeletal muscle derived cells were cultured as described and myoblasts were enriched using magnetic beads. FACS results of different fractions during purification. Top row: Cells before purification. Middle row: Flowthrough, containing $CD56^-$ fibroblasts. Bottom row: Eluate, containing $CD56^+$ myoblasts. Left: Isotype control, labelled with Alexa488 dye. Right: Anti-CD56 antibody, labelled with Alexa488 dye. Percent value are cells in $CD56^+$ gate (R-2). Y-axis: fluorescence. X-axis: forward scatter. Exemplary result from one of the cell batch used in this study.

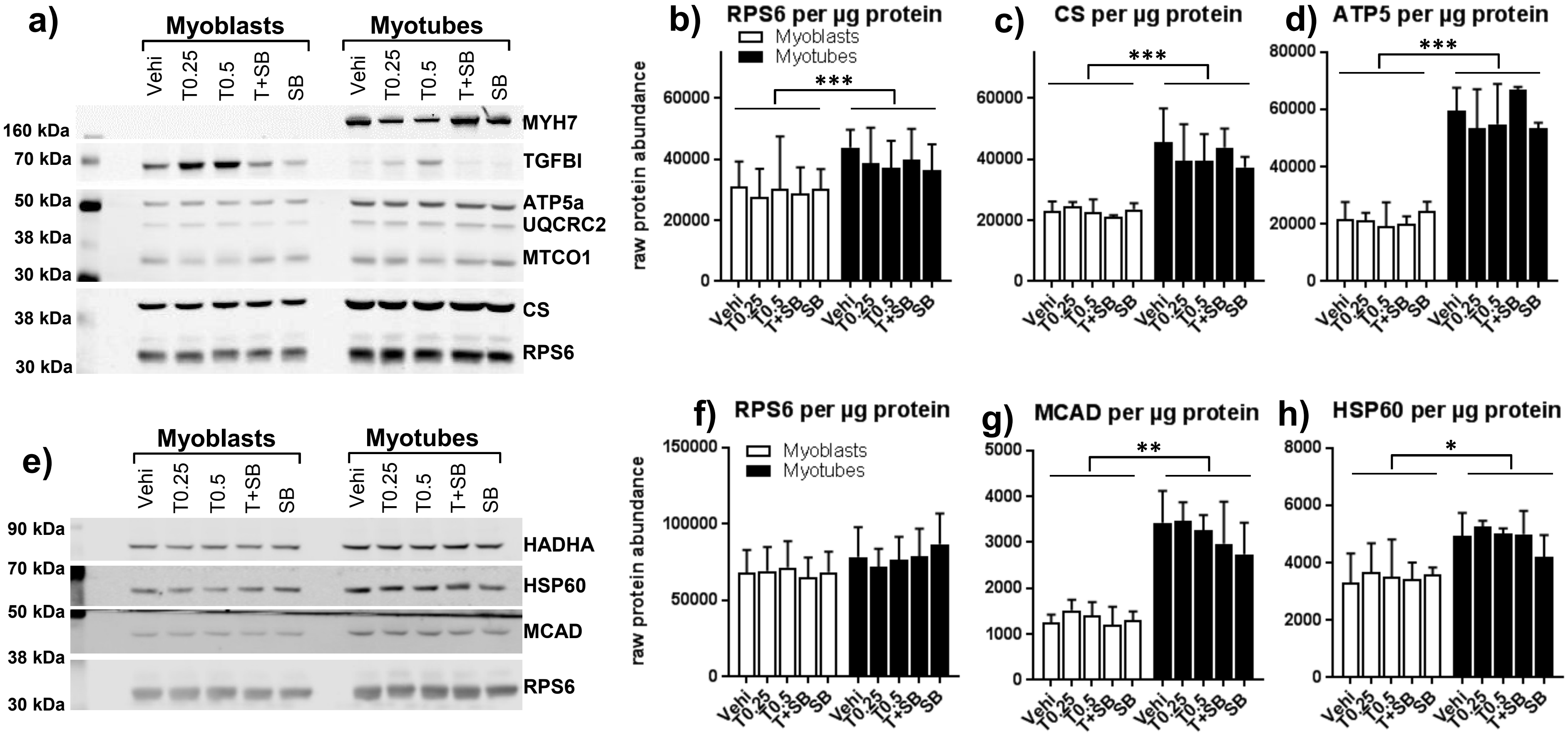

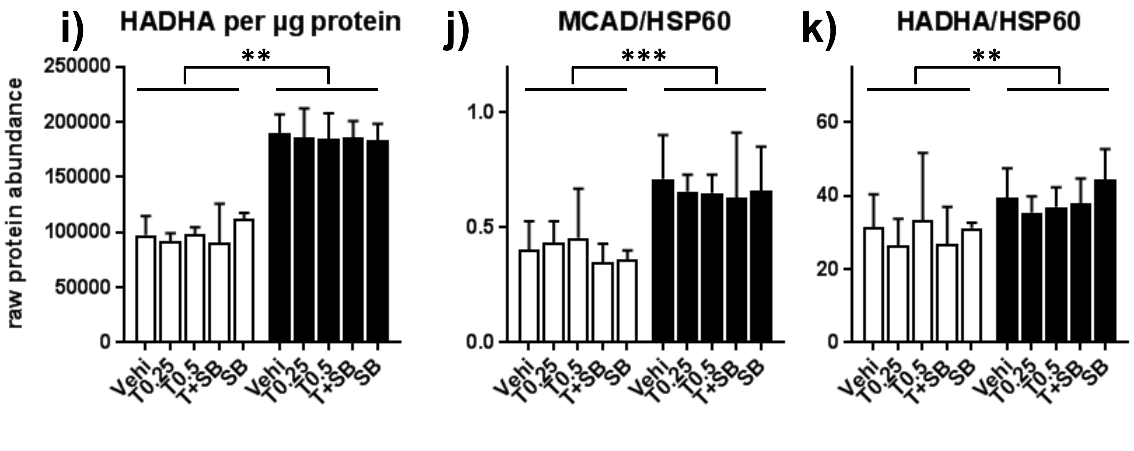

Supplementary Figure S2: Immunoblot data not shown in the main manuscript. Primary human myoblasts and myotubes were treated with TGFβ1 (0.25 or 0.5 ng/ml) or SB431542 (10 µM) for 48 hours and processed according to figure 2. Pre-respirometry protein samples were analyzed by immunoblot. a) Representative immunoblot membrane of cells from one donor using the antibodies as indicated. b) to d) Quantification of RPS6, CS, ATP5, from membrane 1 normalized to protein loaded. Data for MYH7, TGFBI, ATP5a, MTCO1 and UQCRC2 are shown in main manuscript. e) Representative immunoblot membrane of one donor. f) to i) Quantification of RPS6, MCAD, HSP60 and HADHA from membrane 2. j) and k) Data normalized to HSP60. n=3-4, mean ±SD. two-way ANOVA *: $p<0.05$,** $p<0.01$. *** $p<0.001$.

Myoblasts

ETS vs…	OXPHOS	ETS	CS	UQCRC2	MTCO1	ATP5a	TGFBI	UQCRC2/CS	MTCO1/CS	ATP5a/CS
P value	<0,0001		0,0576	0,6030	<0,0001	0,0122	0,0660	0,7293	0,0011	0,0150
R square	0,7560	1,0000	0,2073	0,0173	0,6855	0,3331	0,1958	0,0077	0,4957	0,3167
Correlation	positive		[positive]	none	positive	positive	[negative]	none	positive	positive

OXPHOS vs…	OXPHOS	ETS	CS	UQCRC2	MTCO1	ATP5a	TGFBI	UQCRC2/CS	MTCO1/CS	ATP5a/CS
P value		<0,0001	0,0199	0,6644	0,0002	0,0306	0,7758	0,5070	0,0074	0,1165
R square	1,0000	0,7560	0,2948	0,0121	0,6029	0,2601	0,0052	0,0280	0,3702	0,1469
Correlation		positive	positive	none	positive	positive	none	none	positive	none

Myotubes

ETS vs…	OXPHOS	ETS	CS	UQCRC2	MTCO1	ATP5a	TGFBI	UQCRC2/CS	MTCO1/CS	ATP5a/CS
P value	0,0031		0,1603	0,8479	0,3423	0,1619	0,2568	0,0268	0,3760	0,8400
R square	0,4101	1,0000	0,1125	0,0022	0,0532	0,1117	0,0749	0,2568	0,0464	0,0025
Correlation	positive		none	none	none	none	none	positive	none	none

OXPHOS vs…	OXPHOS	ETS	CS	UQCRC2	MTCO1	ATP5a	TGFBI	UQCRC2/CS	MTCO1/CS	ATP5a/CS
P value		0,0031	0,1501	0,9045	0,0099	0,1198	0,0769	0,0034	0,4242	0,5576
R square	1,0000	0,4101	0,1179	0,0009	0,3311	0,1363	0,1726	0,4054	0,0380	0,0206
Correlation		positive	none	none	negative	none	[positive]	positive	none	none

Supplementary Table S1: Correlation analysis of mitochondrial proteins and respiration data. Primary human myoblasts and myotubes were treated with TGFb1 (0.25 or 0.5 ng/ml) or SB431542 (10 mM) for 48 hours and processed according to figure 2. Pre-respirometry protein samples were analyzed by immunoblot. Correlation analysis for myoblasts (top) and myotubes (bottom). Linear regression modelling using log-transformed data. Representative blot examples can be found in figure S1.
